# Cryotomography of Budding Influenza A Virus Reveals Filaments with Diverse Morphologies that Mostly Do Not Bear a Genome at Their Distal End

**DOI:** 10.1371/journal.ppat.1003413

**Published:** 2013-06-06

**Authors:** Swetha Vijayakrishnan, Colin Loney, David Jackson, Worawit Suphamungmee, Frazer J. Rixon, David Bhella

**Affiliations:** 1 MRC Centre for Virus Research, University of Glasgow, Glasgow, United Kingdom; 2 Biomedical Sciences Research Complex, University of St Andrews, St Andrews, Fife, United Kingdom; Mount Sinai School of Medicine, United States of America

## Abstract

Influenza viruses exhibit striking variations in particle morphology between strains. Clinical isolates of influenza A virus have been shown to produce long filamentous particles while laboratory-adapted strains are predominantly spherical. However, the role of the filamentous phenotype in the influenza virus infectious cycle remains undetermined. We used cryo-electron tomography to conduct the first three-dimensional study of filamentous virus ultrastructure in particles budding from infected cells. Filaments were often longer than 10 microns and sometimes had bulbous heads at their leading ends, some of which contained tubules we attribute to M1 while none had recognisable ribonucleoprotein (RNP) and hence genome segments. Long filaments that did not have bulbs were infrequently seen to bear an ordered complement of RNPs at their distal ends. Imaging of purified virus also revealed diverse filament morphologies; short rods (bacilliform virions) and longer filaments. Bacilliform virions contained an ordered complement of RNPs while longer filamentous particles were narrower and mostly appeared to lack this feature, but often contained fibrillar material along their entire length. The important ultrastructural differences between these diverse classes of particles raise the possibility of distinct morphogenetic pathways and functions during the infectious process.

## Author Summary

Influenza viruses that have been cultivated in the laboratory usually produce particles that are spherical. However, viruses isolated from patients frequently produce long filamentous particles, as well as smaller elliptical particles that we term “bacilliform virions”. Long filaments may be important for cell-to-cell transmission or facilitate release of the smaller particles by disrupting the mucous layer of the respiratory tract. We have used three-dimensional electron microscopy to investigate the structure of influenza virus filaments ‘budding’ from cells. We found that many of the long filaments had a large bulbous head at the end furthest from the cell. Many of these bulbs were empty while some contained tubules that we believe are made of a scaffold-protein M1 that usually lines the inner surface of the viral membrane. Bacilliform virions contain genomes comprised of eight segments of RNA; these are each wrapped up in protein and packaged in an ordered manner. None of the bulb-headed filaments and very few narrower ones had this feature. We hypothesise that the diverse viral structures we have seen suggest distinct assembly pathways and moreover functions. Long filamentous structures that do not appear to contain genomes may combat the immune response or help the smaller virus particles spread.

## Introduction

Each year influenza A viruses cause seasonal epidemics, in which many millions of people worldwide become infected. Pandemic strains emerge periodically as a consequence of the segmented nature of the influenza virus genome that predisposes these viruses to reassortment. Complementary subsets of genome segments from two parental strains come together to form a novel virus with a new antigenic character and possibly altered virulence or species specificity. Influenza A viruses are enveloped, single-stranded negative-sense RNA viruses within the family *Orthomyxoviridae*. The viral envelope is derived from the host cell plasma membrane and bears the glycoproteins haemagglutinin (HA) and neuraminidase (NA) as well as the ion channel protein M2, all of which are critical for virus entry and egress. Beneath the viral envelope is a layer of matrix protein (M1), which is important for virion morphogenesis [1]. The virus interior contains the viral genome, which consists of eight separate RNA molecules [2], [3]. These genome segments are encapsidated by the nucleoprotein (NP) forming eight ribonucleoprotein complexes (RNPs, also termed nucleocapsids), each of which is associated with a viral RNA dependent RNA polymerase (RdRp). Segments one, two and three code for the RdRp proteins (PB2, PB1 and PA respectively), segment four for HA, segment five for NP, segment six for NA, segment seven for M1 and M2, and segment eight for the non-structural protein NS1 and the nuclear export protein (NEP) [4]–[6]. Efficient packing of each segment into a budding virion is directed by specific *cis*-acting RNA sequences. [7], [8].

Influenza virus particles are pleomorphic, showing significant variations in virion morphology among strains characterized as being either spherical or filamentous. Clinical isolates in particular frequently form filaments that can be many microns long when grown in eggs and cell culture [9]–[11]. Metal shadowing electron microscopy experiments showed that filamentous virions sometimes had large varicosities at one end, proposed to bear spherical particles (known as Archetti bodies, [12]). Spherical virion formation is a trait of laboratory-adapted strains, these particles range in diameter between 80 and 170 nm [13]. The process of virus budding and the resulting virion morphology depend on several viral gene products as well as cellular factors. Each envelope-associated gene product (HA, NA, M1 and M2) plays an important role in virion morphogenesis. Virus-like particles can be generated in the absence of M1, suggesting that HA and NA drive budding [14]. HA also plays a critical role in directing M1 to lipid rafts, the site of assembly and release [15]. However while HA and NA are sufficient for budding, M1 appears to be the principal determinant of virion morphology [11], [16], for example a single point mutation (K102A) induces the spherical A/WSN/33 strain to produce filamentous virions [17]. Virion morphology also depends on cell type, a greater proportion of filaments being produced in polarized cells. Disruption of the actin microfilament network is deleterious to filament production [18].

Electron tomography has recently emerged as a powerful tool to investigate pleomorphic virus ultrastructure [19], [20]. Tomograms of resin embedded and frozen-hydrated short rod shaped influenza virions revealed ordered packing of RNPs, with a single central RNP surrounded by a further seven [21]–[24]. It has been suggested that the same ordered arrangement of RNPs is also present at one end of long filaments [21], [22]. This proposition implies that morphogenesis of short and long filaments initiates through the same mechanism of packaging eight genome segments. It also raises the prospect that long filaments may serve a specific function, perhaps in cell-to-cell transmission or possibly in propulsion of progeny virions away from the infected cell as has been reported for vaccinia virus [25].

Here we present a detailed ultrastructural analysis of particles produced by a filamentous strain of influenza A virus (A/Udorn/72 [H3N2]). Immunofluorescent confocal microscopy and cryo-electron microscopy (CEM) were used to image virus infected cells revealing a profusion of long filaments, many of which terminated in a bulbous structure at their leading ends. Cryo-electron tomography (CET) of these Archetti bodies showed that they frequently contained very little material in the terminal varicosities. In those Archetti bodies that did appear to bear contents, the density resembled tubular assemblies of M1 rather than RNPs. CET of purified filamentous virions yielded improved reconstructions of viral filaments showing two distinct forms: short, rod-shaped particles and long narrower ones. Fewer virions with varicosities at the termini were observed suggesting that the majority of Archetti bodies are lost during the purification process.

Overall we found that long filamentous virions exhibited several morphologies and mostly did not appear to contain RNPs at their leading end. Ultrastructural differences observed between classes of filaments raise the possibility of distinct functions and morphogenetic pathways.

## Results

### Imaging of virus budding from cells showed long viral filaments many of which had bulbous heads

**Figure 1:**
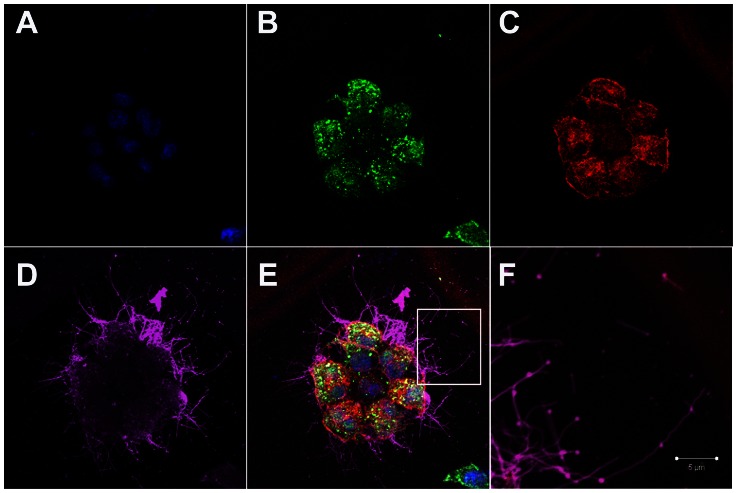
Influenza filaments budding from MDCK cells at 24 hours post-infection. (A) Immunofluorescence shows staining with DAPI (blue), NP (green - B), Actin (red - C) and HA (pink - D). (E) Merged view of channels shown in panels A–D. (F) Close up view of the region indicated by a white rectangle in panel E, revealing budding of filaments of different lengths and morphology and in particular bulbous heads at the distal ends.

**Figure 2:**
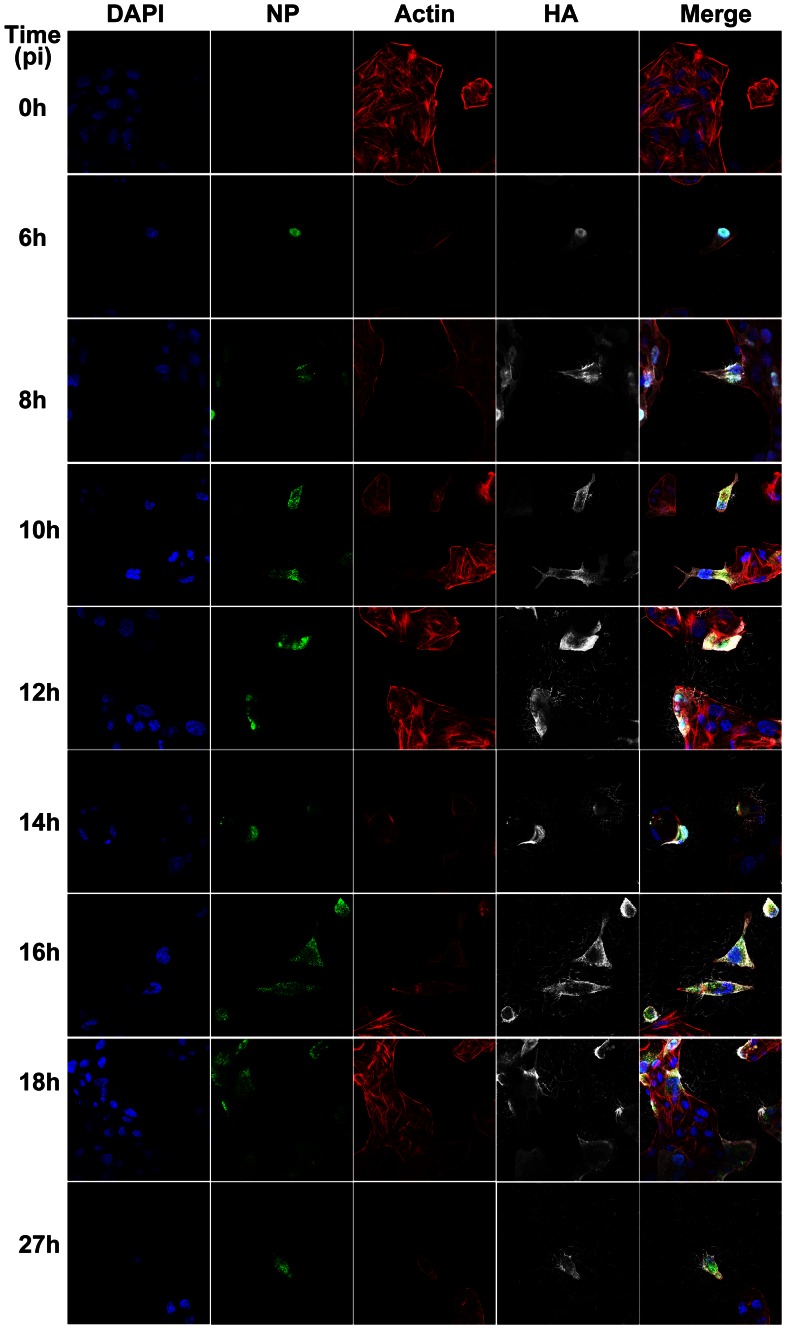
Time course immunofluorescence imaging of filament formation in MDCK cells infected with Influenza A/Udorn/72. DAPI was used to stain cell nuclei (blue) while phalloidin was used to detect actin (red). Monoclonal antibodies were used to detect viral proteins; NP is shown in green and HA is shown in white. Budding virus is seen from as early as 6 hours post infection. From 8 hours we can see long filaments and Archetti bodies at the cell surface.

**Figure 3:**
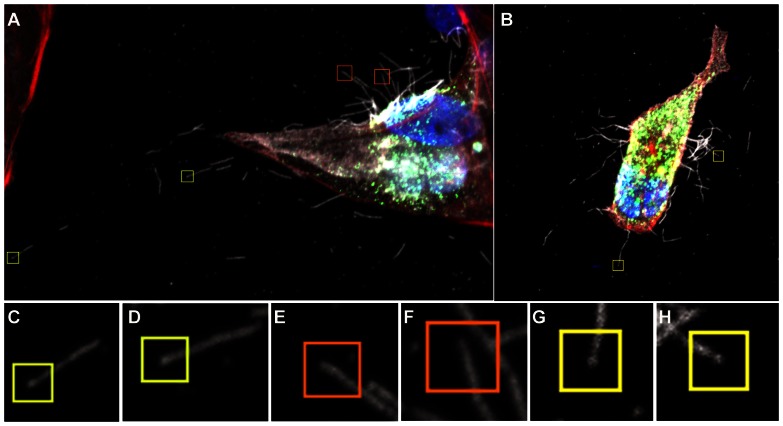
Close up view of filament formation at 8 hours (A) and 10 hours (B) post-infection showing the presence of bulbous termini at the end of some long filaments (C, D) and (G, H) indicated with a yellow box. Many filaments were also seen that did not show stronger fluorescence at their termini, indicating that they were probably not Archetti bodies (E, F orange boxes). See also movie S1.

To achieve an overview of the formation of viral filaments we performed confocal immunofluorescence imaging of MDCK cells infected with influenza virus (A/Udorn/72) at low multiplicity of infection (MOI). This revealed an abundance of viral filaments, many of which had bulbous heads (Archetti bodies). Our images demonstrated the position of these heads to be at the leading (distal) end of the filaments (Fig. 1). Some filaments appeared to have varicosities along their lengths, however such features were not seen in subsequent CET analysis, strongly suggesting that these features were a consequence of several filaments of different lengths lying in close proximity. Viral filaments were extremely long, some measuring greater than 10 µm. A time course was performed revealing the presence of virions as early as 6 hours post-infection (p.i.) (Fig. 2) while filaments and Archetti bodies were seen from 8 hours p.i. (Fig. 2–3, movie S1). Fluorescence imaging of unpermeabilised infected-cells confirmed that the filamentous and Archetti structures seen were not artefacts of preparation (Fig S1). Further control experiments were performed to compare these data with patterns of fluorescence seen in A549 cells infected with Udorn (Fig S2). This revealed that fewer and shorter filaments were produced although the filamentous trait was still in evidence. MDCK cells infected with the spherical Influenza (A/WSN/33) strain on the other hand did not show any filamentous forms (Fig S3).

**Figure 4:**
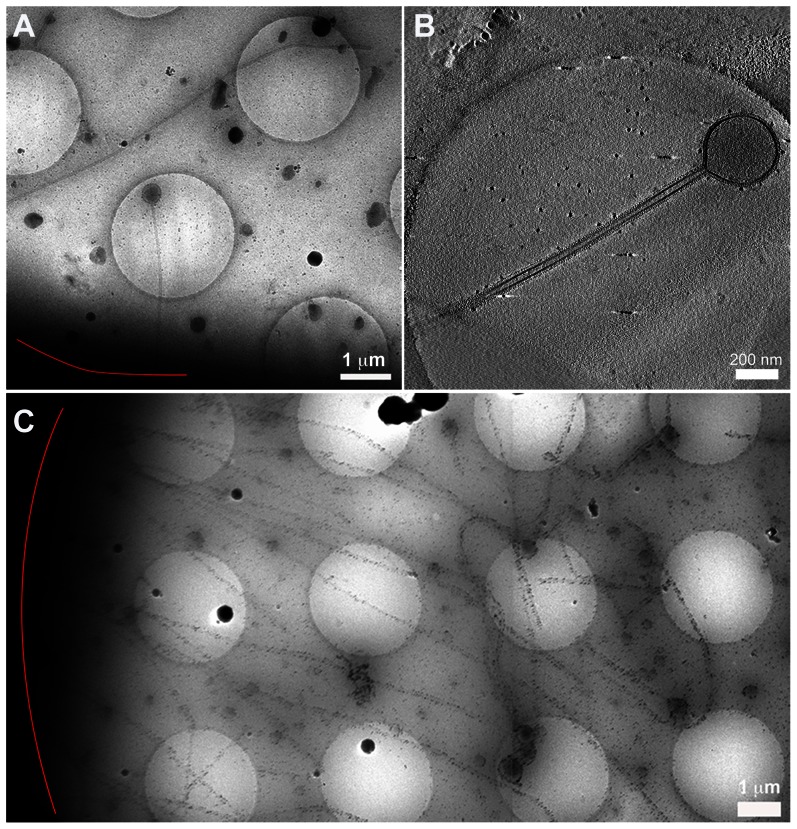
Cryomicroscopy and tomography of influenza A/Udorn/72 infected cells. (A) Low magnification cryomicrograph of a long filament and Archetti body attached to a cell edge (red line). (B) A slice through a tomogram of the Archetti body shown in (A) reveals that the head was largely devoid of content. (C) Filaments over 10 µm long attached to a cell (red line). See also Movie S2 and figures S4 and S5.

**Figure 5:**
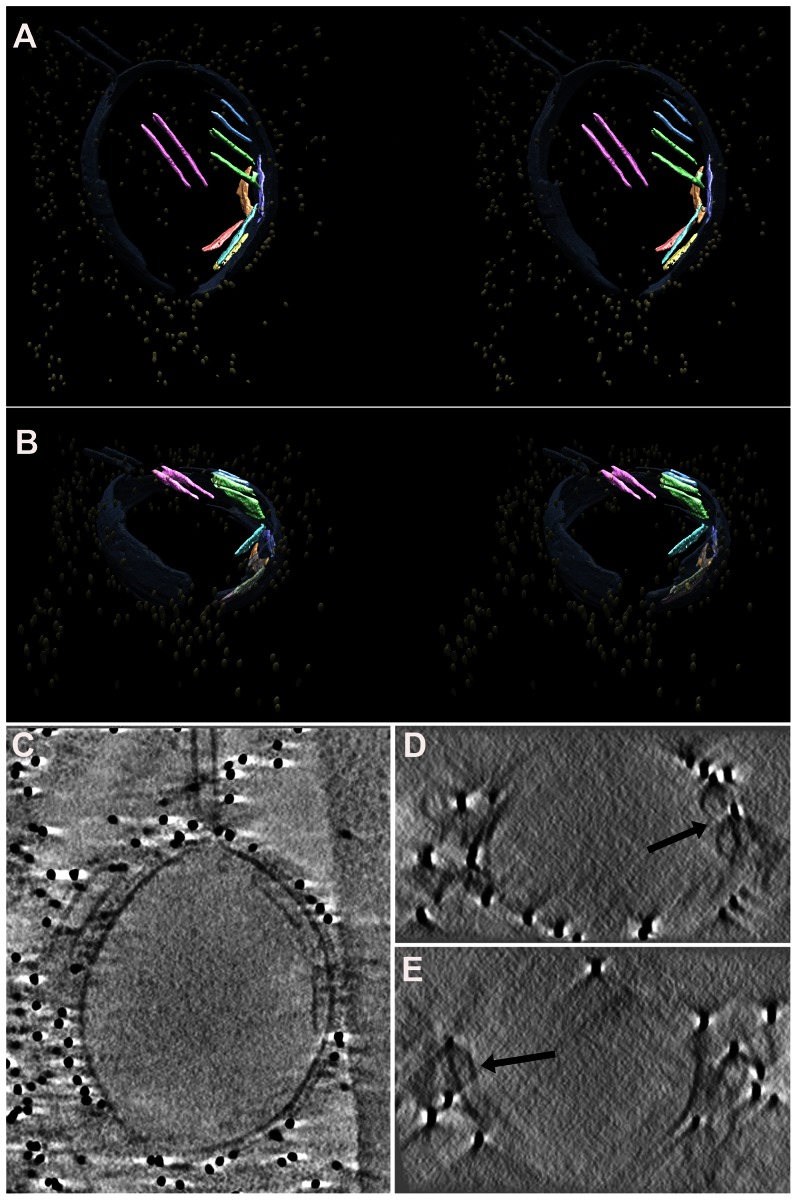
Segmentation of an Archetti body. (A) Stereo images of a segmented and isosurface rendered terminal varicosity, viewed perpendicular to the vitreous ice layer and (B) at 55° to the viewing direction in (A). Density within the bulb showed single or paired sheets (pink, green, orange, light blue, yellow) in close proximity to the membrane (grey). These features were attributed to M1. The gold fiducial markers (mustard) trace the outside edge and extent of the particle. (C) A slice through the same tomogram illustrating the presence of M1 density closely associated with the particle envelope. (D and E) Transverse sections showing that these features appear bracket shaped (black arrow) and are most likely tubes, supporting the view that these are composed of M1. See also Movie S3.

To examine Archetti bodies and other cell associated filamentous structures in more detail and in three-dimensions, we performed CET of infected frozen hydrated cells (Fig. 4, Movie S2). Filamentous structures and Archetti bodies were densely covered in surface spikes confirming their viral origin and had a contiguous matrix layer. The filamentous regions of these particles had a diameter of 74.7±0.78 nm (mean +/− SEM, measured to the tips of the glycoproteins) and extended beyond 10 µm in length (Fig. 4C). Diameters of the bulbous heads ranged from approximately 200 nm to over 550 nm. Of 41 Archetti bodies imaged 25 (61%) were found to be empty while the remaining 16 (39%) had contents within the termini. Segmentation of such particles (Fig. 5A–B, Movie S3) and close inspection of the reconstructed density (Fig. 5C–E) strongly suggested that the contents were tubules formed from M1. These features were seen to be single or paired curved sheets of density (resembling a bracket when viewed in cross-section, Figs. 5D and E). Single sheets lay parallel and closely apposed to the particle envelope (spaced between 25 and 35 nm from the envelope) while paired sheets were 30–35 nm apart. The most likely interpretation of these structures is that they are tubes in which the top and bottom are not well resolved owing to the missing-wedge artefact (features in the z-axis are poorly resolved in electron tomographic reconstructions owing to incomplete sampling caused by the geometry of the transmission electron microscope that prevents tilting the specimen to +/−90°). The missing-wedge also complicated efforts to segment the ‘top’ and ‘bottom’ portions of the viral envelope, however the extent of the bulb was rendered visible by the presence of the gold fiducial markers, revealing that the particle is substantially flattened in the vitreous ice-layer. Furthermore the position of the fiducial markers (which will not have entered the particle) indicates that those putative M1 tubes that are seen as pairs of sheets are also closely apposed to the viral envelope. In all cases the measured internal diameter of the tubes is very similar to the interior diameter of the filamentous particles, supporting our view that these structures are most likely composed of the matrix protein M1.

We have previously reported the presence of tubular matrix derived structures in the paramyxovirus Sendai virus [19]. These structures have been shown to enclose the nucleocapsid in the related measles virus [26]. Our reconstructions of Archetti bodies do not however show evidence of RNPs within these putative M1 tubes.

The filamentous regions of the Archetti bodies sometimes contained sparsely distributed density that could not be attributed to one specific viral protein on the basis of morphology. In addition to being extremely long these filaments were often seen to be flexible (Fig S4A) while others were straight and appeared to be rigid (Figs. 4B, S4A). Such particles sometimes showed fractured apparently open ends (Fig S4B) suggesting that their significant lengths may predispose them to breakage, releasing them from the cell surface. Archetti bodies were however also seen to have pinched off, forming intact particles (Fig S4C–D).

It has proven difficult to visualise the site of budding, as regions of the cell thicker than 500 nm may not be imaged in the cryomicroscope at high-tilt angles owing to the commensurate increase in ice-thickness as tilt angle increases. We have however recorded both low magnification images and tomograms of several filamentous particles that appear to emanate from the cell surface (Fig. 4), some of which are surrounded by cellular processes and vesicles (Fig S5).

**Figure 6:**
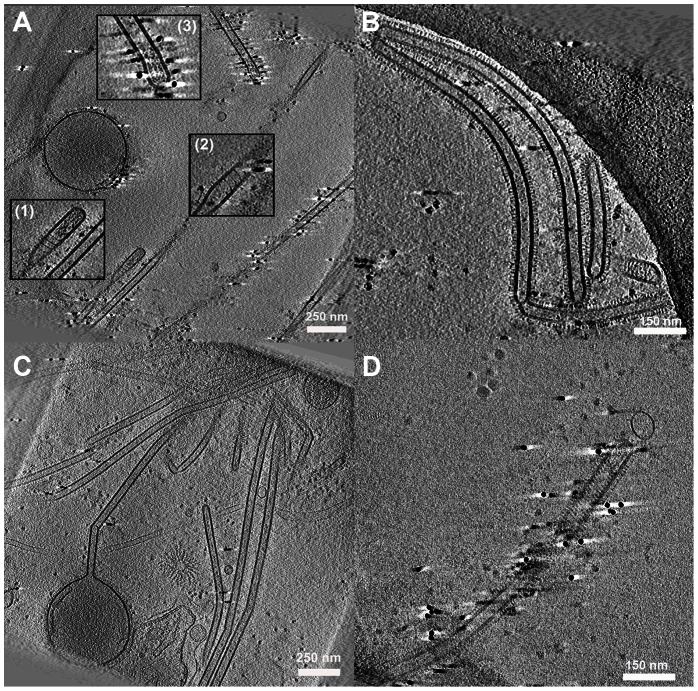
Cell associated long filamentous structures. (A) Tomogram showing a large terminal varicosity and two filamentous particles that appear to contain RNPs at their ends (Insets 1 and 3). An extended helical structure is also seen, possibly M1 (Insets 1 and 2). (B, C) Archetti bodies and the majority of filamentous structures were seen not to have ordered arrangements of RNP at their ends. (D) A filamentous virion containing an extended helical structure that may be M1. See also movie S4.

In addition to Archetti bodies, we saw many filaments that did not terminate with a bulbous varicosity. Some of these filamentous particles had density at their termini reminiscent of the ordered arrangement of genome segments previously described in smaller filamentous particles [21]. The density was not however clearly enough resolved to image the classical ‘seven around one’ arrangement of genome segments in transverse sections (Fig. 6A inset 1 and 3, Movie S4). In these experiments however, we more commonly saw filamentous particles with no distinct density at their termini, rather they had indistinct density along their entire lengths or they were empty (Figs. 6B–C). To evaluate the relative numbers of the various classes of filaments observed we classified 175 long filaments imaged by CET according to the structures seen at their ends. We found that 21.7% of filaments appeared to contain RNPs, 20% of filaments terminated in bulbous Archetti varicosities while 58.3% had no distinct structures at their ends and simply terminated in a hemispherical cap. Thus 78.3% of filaments had no obvious RNP-like structures.

Interestingly in some filaments we saw extended helical density that we hypothesise to be M1 (Figs. 6A inset 1,2, and 6D, Movie S4). The M1 layer is more usually seen as a tightly packed helical array that is closely associated with the envelope but is harder to resolve without prior bromelain digestion of the surface glycoproteins [21]. Negative stain EM experiments of disrupted virions have also unambiguously demonstrated the helical nature of the influenza virus M1 matrix layer [27].

### Cryotomography of purified virions revealed two distinct classes of filamentous particle

**Figure 7:**
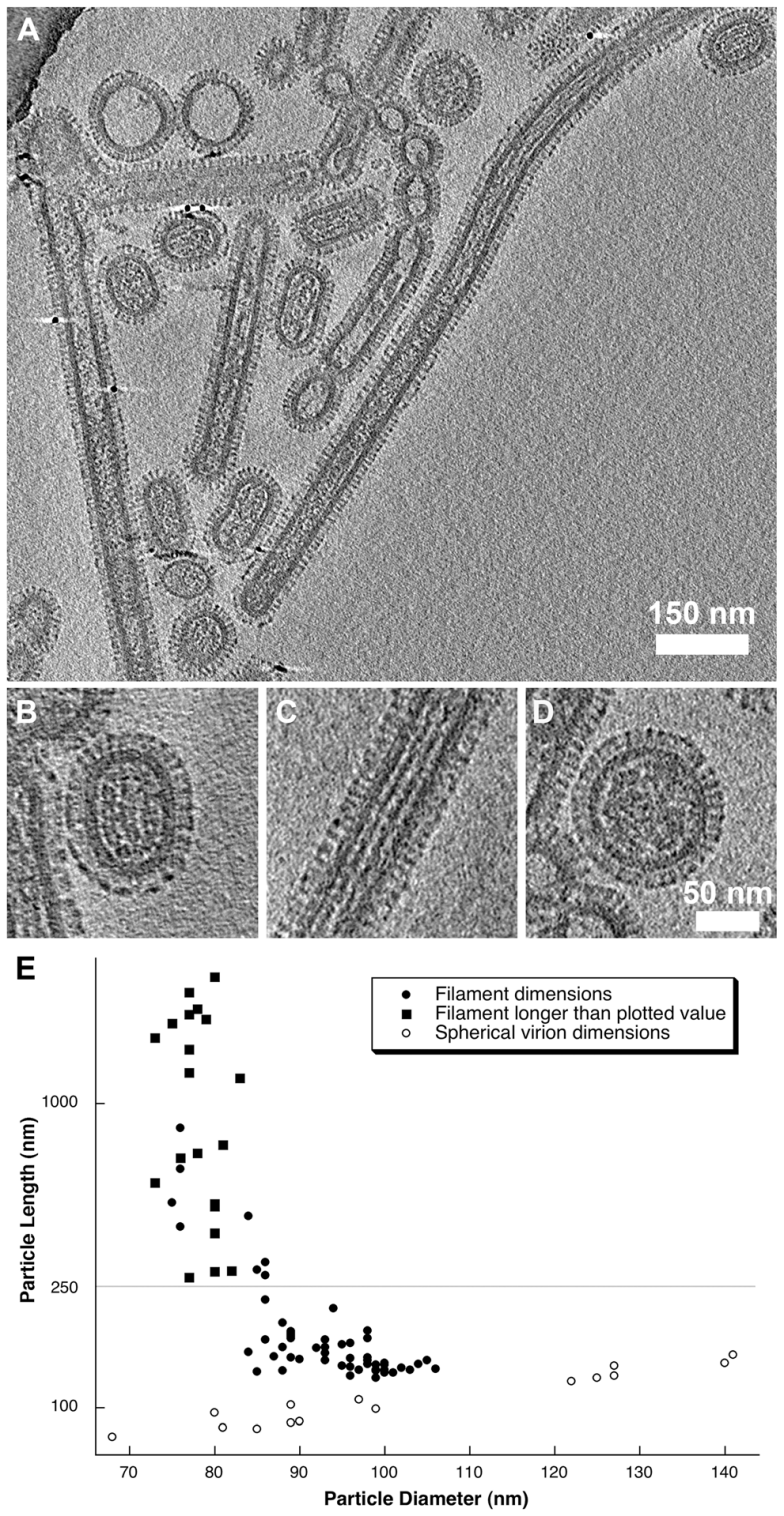
Tomograms of purified A/Udorn/72 virions (A). Three distinct morphologies were observed: short-rods (B), longer filaments (C) and spherical virions (D). Length and diameter measurements from 96 particles were plotted (E) showing that filamentous particles had a narrower diameter compared to the shorter rod-shaped particles (that we term bacilliform virions). Long filaments that extended beyond the field of view were plotted with a filled square. Spherical virion dimensions (those with an axial ratio <1.2) were plotted with a hollow circle. See also movie S5 and figures S6 and S7.

Experiments to image cell associated filamentous structures did not yield data on particles released into the media and in particular smaller virions were only rarely seen (Fig S6A, B). To provide a structural view of all classes of particle produced by infected cells and to compare the morphology of cell-associated filaments with virions and filaments released into the media we performed CET of purified virus particles (Fig. 7A, movie S5). These preparations were predominantly filamentous and very few Archetti bodies were seen (Fig S6C). Some filamentous structures were found to have varicosities along their lengths however these were more irregular in shape and did not resemble the Archetti bodies seen in our study of infected cells (Fig S6D). In our analysis of virus infected cells Archetti varicosities were predominantly seen to be at the termini of budding filaments, confocal imaging on the other hand appears to show filaments with varicosities along their length. Given the greater clarity of the CET data, we conclude that in most cases such features seen in confocal imaging are most likely the result of several Archetti filaments clustering together to give the appearance of a single entity. Very long filaments and Archetti bodies were seen to be fragile and liable to shear, it is likely then that the majority of longer filaments and Archetti bodies were lost during the purification process and were therefore not frequently seen in our study of purified filamentous particles.

Three forms of virion were observed in our preparations: two distinct classes of filamentous particle: short capsule-shaped particles (52% of the population, Fig. 7B) and long filamentous particles (31%, Fig. 7C) as well as small numbers of spherical particles (17%, Fig. 7D). Longer filaments frequently extended to over 2 µm i.e. beyond the field of view. Our classification of filamentous particles into distinct groups was based on an analysis of their dimensions in four tomograms of one virus preparation.

96 particles were selected and classified as either filamentous or spherical (having an axial ratio <1.2). Diameter and length measurements were made on the dataset and are plotted in Fig. 7E. For filamentous particles that extended out of the field of view the length of the proportion of the filament that was imaged was plotted. Several filaments that appeared concatenated, perhaps having failed to pinch-off, were measured as a single virion. Plotted particle dimensions clearly showed that short filaments had a larger diameter than the longer structures. To determine the statistical validity of this observation, particles were grouped according to whether they were longer or shorter than 250 nm. Student's t-test confirmed that the two filamentous classes had significantly different mean diameters (t = 13.745, d.f = 77, p<0.0001). The shorter class was found to have a mean (+/− SD) diameter of 94.9 (+/−5.6) nm, while longer filamentous particles had a mean diameter of 78.8 (+/−3.6) nm (measured to the tips of the glycoproteins). We introduce the term “bacilliform” to refer to the shorter class of filamentous particles and distinguish them from the long filaments. A strict definition on the basis of a single measurement is not helpful however owing to an overlap in dimensions between filaments of intermediate length and diameter. Consideration of morphological differences between these two kinds of particles is also important therefore.

**Figure 8:**
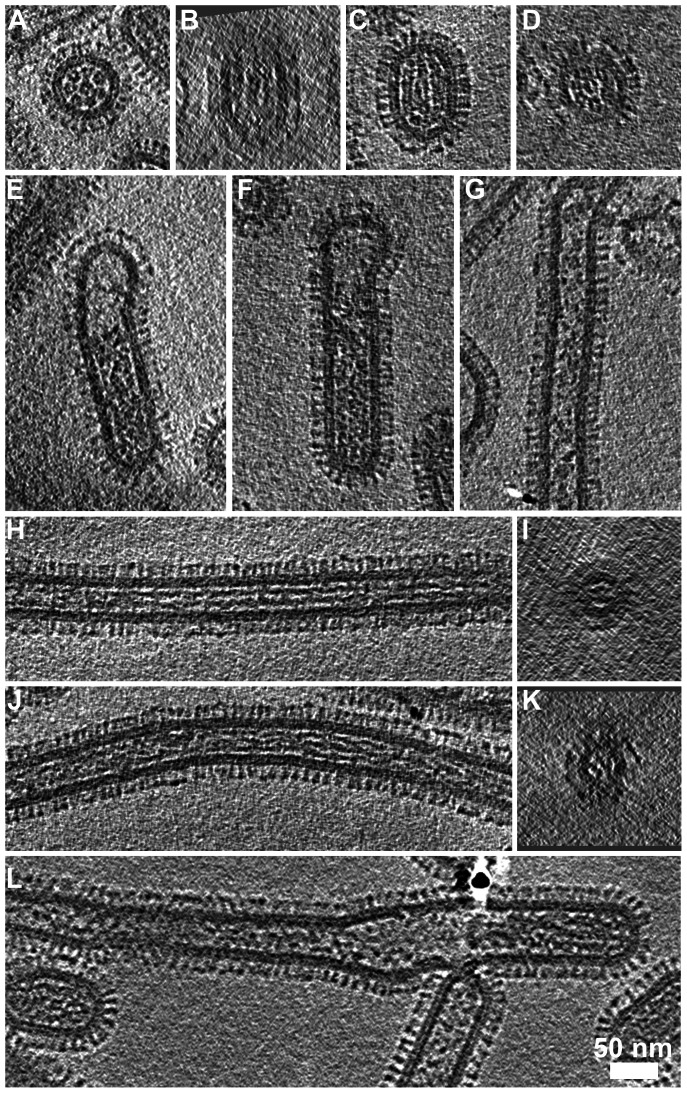
RNP arrangement and other internal components in the various classes of virions. (A) Transverse sections through bacilliform particles revealed the characteristic arrangement of RNPs. (B) Longitudinal section of the particle in (A) showed three RNPs lying side-by-side. (C) Such views were commonly observed with particles oriented parallel to the ice layer and transverse sections through these particles (D) showed the 7+1 arrangement of RNPs. (E, F) Longer bacilliform particles had RNPs at one end while longer filaments (G) were sometimes sparsely packed but more frequently contained fibrillar material along their entire length (longitudinal and transverse sections are shown H–K). In some cases the internal density appeared as straight rods (H, I) while in others it appeared to be wound around itself (J, K). Tomogram sections perpendicular to the ice layer were harder to interpret owing to the missing wedge; an imaging artefact associated with tomographic data acquisition (B, D, I and K). (I and K) Sections through the narrow filaments do not show the RNP morphology seen in comparable views of bacilliform particles (A and D). This feature was infrequently seen at the termini of cell-associated filaments and in our data seen only once in tomograms of purified filaments (L). See also movie S5.

Close inspection of the filamentous structures revealed major morphological differences between the long narrow filaments and bacilliform particles. Transverse sections through tomograms of bacilliform particles showed a pattern of RNP packaging that adheres to that previously described [13], [22]. Eight segments were arranged in an orderly fashion: with a single RNP at the centre of the virion and a further seven arranged around it (Fig. 8A, Movie S6). In bacilliform particles viewed in longitudinal section three segments were sometimes seen lying side by side (Figs. 8B–C). Such views were readily observed in our data, as the majority of particles were oriented with their long axis parallel to the ice layer. Transverse sections through these reconstructions showed that these particles also had the characteristic RNP arrangement (Fig. 8D). RNPs measured between 10 and 14 nm in diameter with a clear channel running along their centre.

Small numbers of longer bacilliform particles (190–300 nm with a diameter of approximately 85–90 nm) appeared empty at one end and contained RNP density that may correspond to a single complement of genome segments (Figs. 8E and 8F). Similar images have led to the suggestion that very long filamentous virions have a single genome copy at one end [21], [22]. However, internal structures in long filaments were distinct and somewhat harder to interpret in our data. Density frequently extended along significant proportions of these particles and was fibrillar in appearance, measuring approximately 5 nm in diameter with no central channel. Other filaments were only sparsely packed or contained clumps of density (Fig. 8G). In particles that were densely packed, fibrillar density sometimes appeared as long straight rods (Fig. 8H), or appeared wound around itself (Fig. 8J). Transverse sections through these filaments reveal that the fibrillar contents clearly do not correspond with the ordered arrangement of RNPs seen in bacilliform virions (Compare Figs. 8I and K with 8A and D). Indeed those long filaments having an ordered complement of genome segments that were rarely observed in studies of infected cells were even more infrequently seen in tomograms of purified filaments (One seen - Fig. 8L). While many long-filament termini were imaged, the great majority of which were devoid of RNPs, fewer tomograms of filaments were available in which both ends were seen. Those that were imaged however did not appear to contain RNPs and were either empty or had fibrillar contents along their entire length (Fig S7).

## Discussion

We have examined influenza A/Udorn/72 filament formation by CET and immunofluorescent confocal microscopy. Confocal microscopy revealed the presence of a variety of filamentous forms: long straight filaments, flexible filaments and Archetti bodies. CET demonstrated that filaments produced in MDCK cells frequently do not contain ordered RNPs at their distal ends and more often terminate with empty ends or Archetti varicosities. These data represent the first structural analysis of influenza virus filaments budding from the host cell under near native conditions. Early metal shadowing TEM studies of filamentous virions produced in eggs described Archetti bodies and proposed that they might bear spherical virions [12], [28]. Our data refute these suggestions and reveal the absence of RNPs or spherical virions in the terminal varicosities of Archetti bodies. It is unclear from our analysis whether these particles bud from the cell surface as bulbous structures or whether the terminal varicosity forms after budding. The presence of tubular assemblies that we attribute to M1 in many Archetti termini suggests that the latter may be the case. However, the envelope measures approximately 12 nm thick in this region, similar to measurements made of the envelope/matrix component of purified particles, budding filaments and empty Archetti bodies. This suggests that the presence of M1 tubes might not be the result of them having detached from the inner surface of the envelope, an occurrence that could result in loss of filamentous form. We frequently saw density within filaments that appears to be a second layer of M1, such as the helical assemblies shown in figure 6 and inclusions within varicosities (Fig S6D). It is possible then, that concentric layers of M1 may be a common feature of influenza A filaments. Owing to the missing wedge we are unable to unambiguously image the entire envelope of the Archetti varicosity however. Thus the origin of the putative M1 tubes cannot be conclusively proven and further experimentation is required to establish the origin of these structures and the morphogenetic pathway of Archetti particles.

As in our study of budding virus, imaging of purified virus also showed the presence of long filamentous particles. Previous structural studies of purified influenza A/Udorn/72 virus imaged filaments of intermediate length (∼500 nm) and did not highlight the fundamental differences in internal features between filaments and bacilliform particles that we have observed [21]. Our data clearly showed the presence of fibrillar material running along the filament interior and longer filaments containing RNPs were very rare. We saw very few spherical particles and the majority of smaller particles would be better described as obloid, prolate or bacilliform. These particles contained a well-ordered arrangement of RNPs when viewed in transverse and longitudinal sections that appeared similar to those previously described and have been shown to be supercoiled circularized nucleocapsids [21]–[24], [29]–[31]. Harris *et al.* observed dense ‘solenoid shaped’ material proposed to be nucleocapsids in their CET study of spherical influenza A virions [13]. Similar density observed in filamentous virus by Calder *et al.* was attributed to M1 however [21]. This illustrates the difficulties associated with attributing density in tomograms to specific components on the basis of morphology and in the absence of firm biochemical evidence. Likewise we have encountered difficulties in our interpretation of density within very long filaments and have yet to determine the origin of material seen in purified and cell associated filaments. It should be noted that in our experiments we have visualised different influenza filament populations in purified preparations than in virus-infected cells. The fibrillar density seen in purified particles is more clearly resolved than interior density in cell-associated filaments. Thicker ice in the latter experiments may reduce the contrast of these features; indeed surface glycoproteins are also less well resolved. Alternatively this may reflect genuine differences in filament composition.

Long filamentous particles had a narrower diameter than the bacilliform particles and there was frequently no evidence of a well-ordered arrangement of RNPs inside. Our findings indicate that the longer filamentous forms do not in the main simply represent elongated versions of the shorter bacilliform particles. Various classes of filaments are present and these might assemble according to distinct morphogenetic pathways. Both filaments and bacilliform virions have a regular cylindrical shape, capped at each end by a hemisphere, or in the case of Archetti bodies, with a bulbous head at one end. There is considerable evidence that the filamentous phenotype is controlled by M1 [11], [16], [17], moreover preservation of the filament morphology correlates with an intact M1-layer. It seems plausible therefore, that formation of one or other class of virion may be controlled by the oligomerisation of M1 at the site of virion assembly, where the resulting curvature of the leading end might be influenced by a number of factors including M2 [32] and the presence or absence of eight RNPs. This latter possibility provides perhaps the simplest explanation of the various forms seen. If viral proteins were capable of initiating assembly at the plasma membrane in the absence of RNPs this may result in a smaller radius of curvature and consequently a narrower filament. The proximal end of RNPs in the budding virion may also be required to initiate pinching off, with the absence of this signal therefore leading to long filament formation. The smaller radius of curvature or absence of RNPs at the distal end may also result in a less robust structure, prone to loss of integrity and formation of the Archetti varicosity (although we see no evidence of loss of the envelope M1 layer). Small numbers of long filaments that contain RNPs might also be formed as a consequence of a failure in the pinching off process, leading to a switch to the alternative helical packing of M1 necessary for the formation of the narrower diameter long filament.

Despite clinical isolates frequently exhibiting the filamentous phenotype, the role played by these long filaments in the infectious process is yet to be established. They have been suggested to be involved in cell-to-cell transmission [18]. Clearly virions that can be greater than ten microns in length could not initiate infection via receptor-mediated endocytosis. It has recently been shown that filaments can however enter cells by macropinocytosis [33]. Recent work has also highlighted possible advantages of the filamentous shape over spherical in the context of effective transport and trafficking to the respiratory epithelium [34]. The various functions of the diverse filaments we observe may be resolved upon identification of the density at the particle interior. The small number of filaments that comprise an ordered complement of RNPs at their leading end may indeed play a role in cell-to-cell transmission. There is also evidence to suggest that filaments may contain multiple sets of RNPs; early studies showed higher infectivity in filament preparations in comparison to spherical virions while UV inactivation experiments suggested that filaments are polyploid [16], [35], [36]. Our data do not refute this however it is very difficult to create pure preparations of long filaments to verify these data. If the material in these particles were found not to be RNPs this would point to an alternative role, perhaps in virus pathogenesis. Immunoglobulin A (IgA) is highly expressed in the respiratory tract and plays a key role in resistance to influenza infection [37]. It is possible that those long filaments and/or Archetti bodies with no genome segments, being studded with significant quantities of HA might act as a “decoy” for the host immune response, binding antibody and helping the infectious virions to evade neutralisation. It is also conceivable that due to their extreme lengths they could physically disrupt the mucociliary layer in the respiratory epithelium, thereby facilitating rapid spread of infectious bacilliform particles.

We have shown that the filamentous Udorn/72 strain of influenza A virus produces a variety of filamentous particles and Archetti bodies, the majority of which do not contain ordered RNPs at their leading end and may have a unique role to play during viral infection. The functional significance of these diverse structures remains to be confirmed. Future studies employing human airway model systems may provide further insights and a more biologically relevant view of the role these particles play in the infectious process and viral pathogenesis.

## Materials and Methods

### Virus propagation

The H3N2 strain influenza A/Udorn/72 was cultivated in MDCK cells. Cells were grown to confluence at 37°C in Dulbecco's modified Eagle's medium (DMEM) supplemented with 10% foetal calf serum (FCS). 3×10^8^ cells were infected at a multiplicity of infection (MOI) of 0.001. Following an incubation period of 1 h, the media was replaced with serum-free DMEM containing 2.5 µg/ml N-acetyl trypsin (NAT, Sigma) followed by incubation at 37°C. 36 hours post-infection (p.i.), the supernatant was harvested and clarified by centrifugation at 2500 rpm for 5 min. Following a second clarification step (10,000 rpm for 30 minutes), virus was pelleted by centrifugation onto a 30% sucrose cushion in NTE buffer (1 mM EDTA 10 mM, 150 mM NaCl, Tris-HCl, pH 7.5) at 25,000 rpm for 2.5 h. The pellet was then resuspended in 250 µl NTE and run through a continuous sucrose gradient (30%–60%) at 25,000 rpm for 2.5 h. Finally, banded virus was collected and centrifuged at 31,000 rpm for 2 h. The pelleted virus was resuspended in 100 µl of NTE buffer.

### Confocal microscopy

Confluent monolayers of MDCK cells grown on cover slips were infected at an MOI of 0.6. Cells were then fixed 24 h p.i. with 4% formaldehyde/2.5% Triton X-100 in PBSA for 30 min. They were then washed three times with PBS, followed by blocking in sheep serum or rabbit serum for 1 h. Labelling was then performed by incubating cells for 1 h at room temperature with primary antibodies. Unbound antibody was removed by washing three times with PBSA prior to incubation for 30 min at room temperature with fluorescent-tagged secondary antibodies. Finally cells were washed three times with PBSA and mounted using ProLong Antifade plus DAPI reagent (Invitrogen, UK). For the time course experiment, infected MDCK cells (moi 3) were fixed at various time points; 6 h, 8 h, 10 h, 12 h, 14 h, 16 h, 18 h and 27 h. Infected MDCK cells fixed at 27 h with only 4% formaldehyde served as a non permeabilised control. Controls were also carried out by infecting A549 cells with H3N2 Udorn (moi 3) and MDCK cells with H1N1 WSN (moi 5) followed by fixation with 4% formaldehyde/2.5% Triton X-100 13 h p.i. Labelling for the time course and control samples were performed as detailed above. All samples infected with H3N2 Udorn were immunolabelled for the H3 haemagglutinin with a mouse monoclonal antibody raised against A/X-31/1968 H3N2 virus, kindly provided by Prof. John Skehel (NIMR, London), while the H1N1 WSN samples were labelled for H1 with a mouse monoclonal antibody raised against A/PR/8/34 H1N1, kindly provided by Prof. Paul Digard (Edinburgh). H3 and H1 were detected with a rabbit anti-mouse Alexa Fluor 633 (Invitrogen, UK). Nucleoprotein (NP) labelled using a mouse monoclonal antibody (Abcam, UK) was detected using a sheep anti-mouse-FITC conjugate (Sigma, UK). Phalloidin Alexa Fluor 568 (Invitrogen, UK) was used to stain actin. Immunofluorescent imaging was carried out with Zeiss LSM510 Meta and LSM710 laser confocal microscopes.

### Infection of cells grown on grids

Gold 200 mesh TEM grids with holey carbon support film (Quantifoil Micro Tools GmbH, Jena, Germany) were sterilized with ethanol and then coated with laminin overnight in glass-bottomed dishes (MATTEK Corporation Inc, USA). Grids were then washed in water and seeded with 100,000 MDCK cells per dish in DMEM media supplemented with 10% FCS. They were then incubated overnight at 37°C. Cells were infected at an MOI of 0.6 for 1 h at 37°C. The media was then replaced by serum free DMEM supplemented with 2.5 µg/ml NAT and incubated for 19 h.

### Electron Microscopy and image reconstruction

For cryo imaging of purified virus, preparations were mixed with 10 nm colloidal gold (British Biocell International, Cardiff, UK) in a ratio of 1∶3 v/v. A 5 µl aliquot was applied to freshly glow-discharged Quantifoil holey carbon support films (R2/2 200 mesh copper grids - Quantifoil Micro Tools GmbH, Jena, Germany), blotted and frozen by plunging into liquid ethane as previously described [38].

For cryo imaging of virus infected cells grown on Quantifoil EM grids 15 nm colloidal gold (British Biocell International, Cardiff, UK) was added in a ratio of 1∶3 v/v. Grids were then blotted and frozen by plunging into liquid ethane.

Tilt-series imaging was performed on a JEOL 2200FS energy-filtering transmission electron microscope equipped with a Gatan Ultrascan 4 k×4 k CCD camera and a Gatan 914 high-tilt cryo-stage. The microscope was operated at 200 kV and zero-loss energy filtered imaging with a slit-width of 30 eV was used to enhance image contrast. Tilt series were recorded using the SerialEM software package [39]. Images were acquired at two-degree increments from −70° to +70° between 10,000× and 20,000× magnification for cells on grids and at 20,000× or 40,000× magnification for purified virus. Images were recorded with two-times binning, corresponding to a pixel sizes ranging from 21.2 to 5.4 Å/pixel in the specimen. The target defocus was set to between 4 and 6 µm under-focus and the electron dose ranged from 83 e/Å^2^ to 100 e/Å^2^ per tilt-series. Tomograms were calculated and visualized using the IMOD software package [40]. Reconstruction was performed using weighted back projection followed by denoising using non-linear anisotropic diffusion. Figures were prepared by averaging 10 tomogram sections using IMOD's 3dmod slicer routine. Segmentation was performed manually using Amira (Visual Sciences Group).

## Supporting Information

Figure S1 Immunofluorescent confocal imaging of unpermeabilised MDCK cells infected with Influenza A/Udorn/72 virus, showing that filaments and Archetti bodies (arrows) are not an artefact of preparation.
(TIF)

Figure S2 Confocal imaging of Influenza A/Udorn/72 infection in A549 cells shows that fewer filaments are produced in this cell line compared with MDCK cells, however the filamentous phenotype is still evident. The top row images show uninfected cells (UI), the bottom row shows A549 cells infected with Influenza A/Udorn/72 (Ud).
(TIF)

Figure S3 Comparison of immunofluorescence patterns in MDCK cells infected with Influenza A/Udorn/72 (an H3N2 filamentous virus) and A/WSN/33 (an H1N1 spherical virus). While abundant filaments are seen in the Udorn infected cells, this feature is not seen in the WSN infection.
(TIF)

Figure S4 Cryo Electron Microscopy (A, B) and Cryo Electron Tomography (C, D) of Archetti bodies. Archetti bodies budding from cells were seen to be very long (>10 µm) and were straight (white arrows) and/or flexible as denoted by the black arrows (A). Their extreme lengths predisposed them to shearing and breakage into smaller rods, shown by the white circles (B). Archetti bodies were also seen to have budded from the cell surface resulting in particles with large varicosities at one end and normal hemispherical caps at the other (C, D).
(TIF)

Figure S5 Cryo Electron Microscopy of budding filaments and Archetti bodies at the cell surface. Cell associated filaments and Archetti bodies close to the cell edge (red line) were seen to be surrounded by vesicles and cell processes (A, B).
(TIF)

Figure S6 Visualising the pleomorphic structures of influenza A filaments and virions. In most tomograms of virus infected cells, small virions were not observed as they were most likely suspended in the culture media and did not adhere to the carbon support film. One tomogram was however recorded in which long filaments and Archetti bodies were seen (A) as well as some smaller virions (B). A low magnification (4000×) cryo image of purified virus reveals the extent of pleomorphism showing long filaments and a small number of Archetti bodies (white circles - C). Tomograms of purified virus however did not show Archetti bodies resembling those we saw in virus infected cells, rather filaments with varicosities along their lengths were seen that were less regular and frequently contained vacuolar structures (D).
(TIF)

Figure S7 Cryotomography of purified influenza A filaments mostly did not have RNPs at their termini. Filaments in which both ends were visible were not commonly seen in tomograms owing to their great lengths however. Where such filaments were observed, the ends (red boxes) were not found to contain obvious RNP like density (A–C).
(TIF)

Movie S1 Movie to show confocal imaging of Archetti bodies at 18 hours post-infection. DAPI was used to stain cell nuclei (blue) while phalloidin was used to detect actin (red). Monoclonal antibodies were used to detect viral proteins NP is shown in green and HA is shown in white.
(MOV)

Movie S2 Movie to show serial sections through the z-axis in a tomogram of an Archetti body budding from MDCK cells (also shown in Figure 4).
(MOV)

Movie S3 Movie showing a cross-eyed stereo image of a segmented Archetti body containing single or paired sheets of density (pink, green, orange, light blue, yellow) in close proximity to the membrane (grey). These features were attributed to M1. The gold fiducial markers (mustard) trace the outside edge and extent of the particle. The tomogram is also shown as serial sections through the reconstructed density.
(MOV)

Movie S4 Series of movies showing serial sections through tomograms of cell-associated filamentous particles. Archetti bodies are shown that contain putative M1 tubules. Helices that we attribute to M1 are also shown within long filaments. Long filaments are shown in which RNPs are seen at the distal ends while other filaments do not contain this feature.
(MOV)

Movie S5 Movie to show a tomogram of purified Udorn H3N2 virus. A single section through this reconstruction is presented in figure 5A.
(MOV)

Movie S6 Movie to show the 7+1 arrangement of RNPs in bacilliform particles and fibrillar density in longer filamentous particles.
(MOV)
