# Supplementary figures and images for: Cryotomography of Budding Influenza A Virus Reveals Filaments with Diverse Morphologies that Mostly Do Not Bear a Genome at Their Distal End
Source: PLoS Pathog. 2013 Jun 6;9(6):e1003413. doi: 10.1371/journal.ppat.1003413 (PMC3675018; doi:10.1371/journal.ppat.1003413)

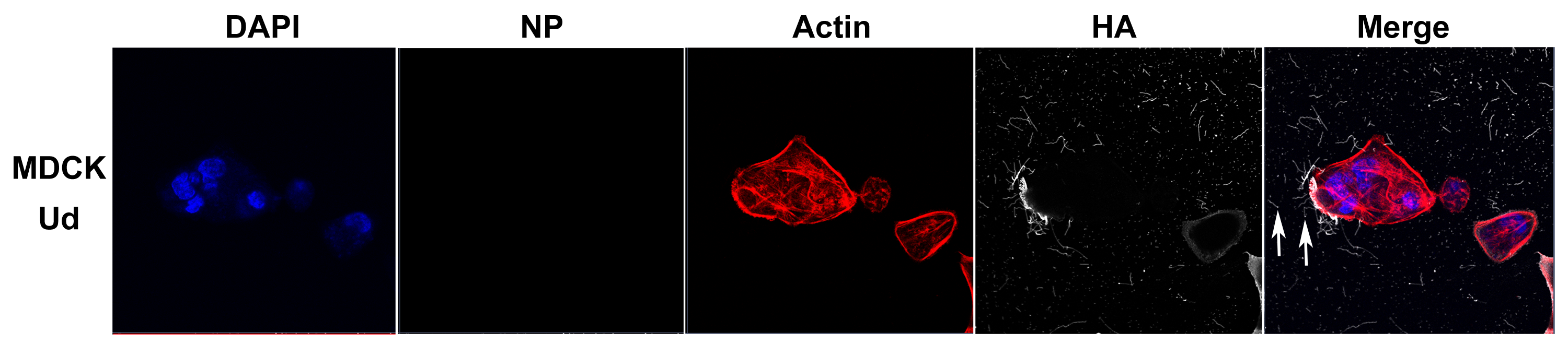

Supplement: Figure S1 — Immunofluorescent confocal imaging of unpermeabilised MDCK cells infected with Influenza A/Udorn/72 virus, showing that filaments and Archetti bodies (arrows) are not an artefact of preparation. (TIF) [file ppat.1003413.s001.tif]

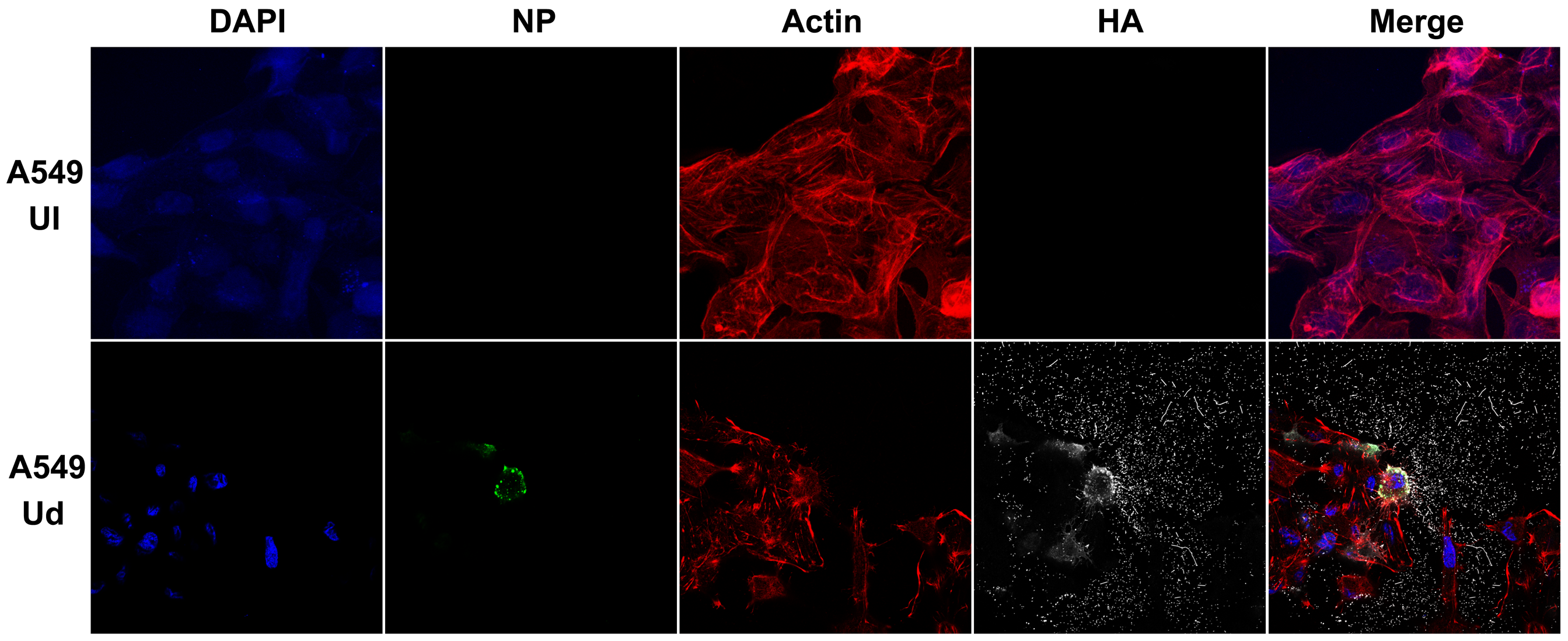

Supplement: Figure S2 — Confocal imaging of Influenza A/Udorn/72 infection in A549 cells shows that fewer filaments are produced in this cell line compared with MDCK cells, however the filamentous phenotype is still evident. The top row images show uninfected cells (UI), the bottom row shows A549 cells infected with Influenza A/Udorn/72 (Ud). (TIF) [file ppat.1003413.s002.tif]

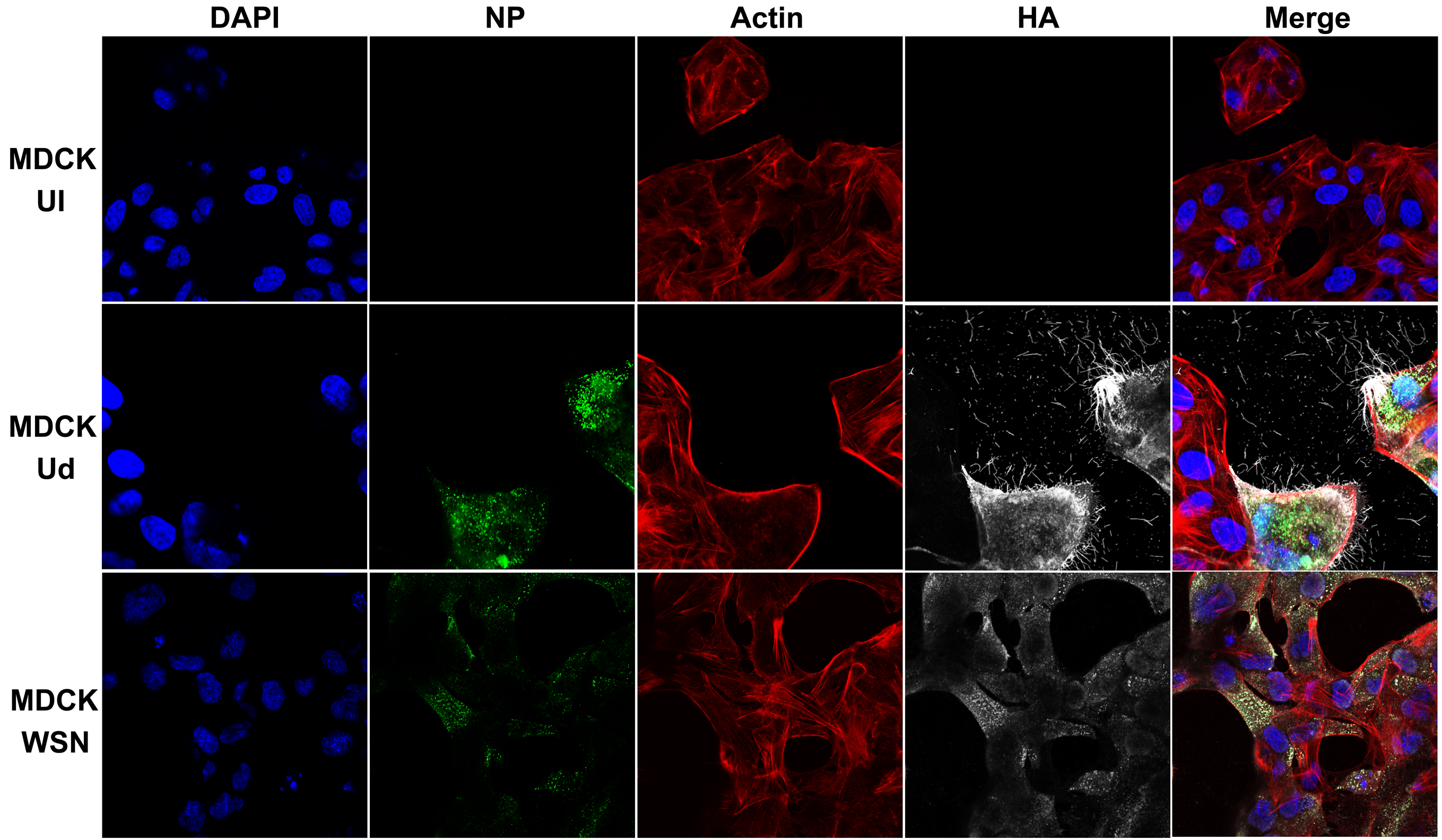

Supplement: Figure S3 — Comparison of immunofluorescence patterns in MDCK cells infected with Influenza A/Udorn/72 (an H3N2 filamentous virus) and A/WSN/33 (an H1N1 spherical virus). While abundant filaments are seen in the Udorn infected cells, this feature is not seen in the WSN infection. (TIF) [file ppat.1003413.s003.tif]

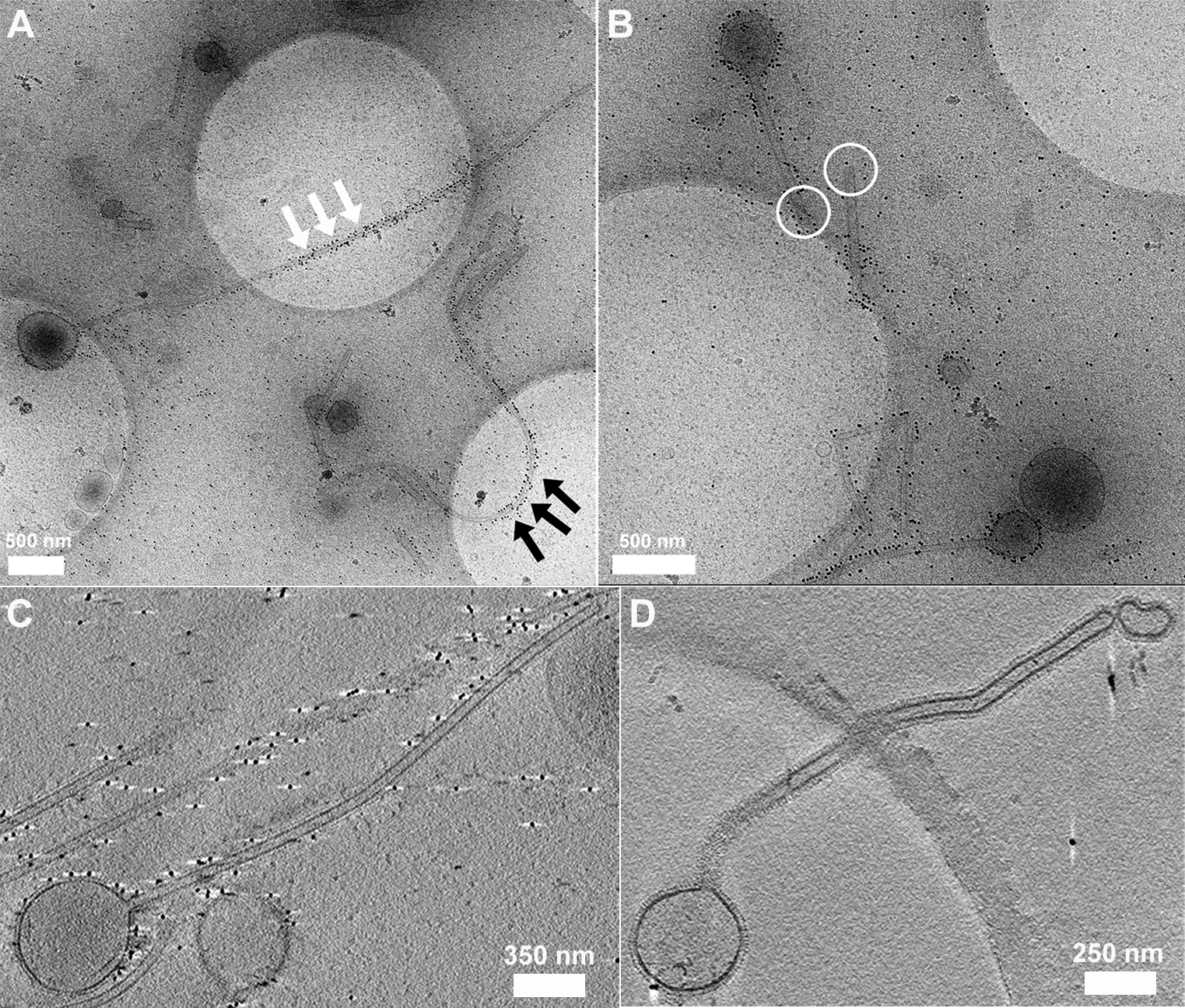

Supplement: Figure S4 — Cryo Electron Microscopy (A, B) and Cryo Electron Tomography (C, D) of Archetti bodies. Archetti bodies budding from cells were seen to be very long (>10 µm) and were straight (white arrows) and/or flexible as denoted by the black arrows (A). Their extreme lengths predisposed them to shearing and breakage into smaller rods, shown by the white circles (B). Archetti bodies were also seen to have budded from the cell surface resulting in particles with large varicosities at one end and normal hemispherical caps at the other (C, D). (TIF) [file ppat.1003413.s004.tif]

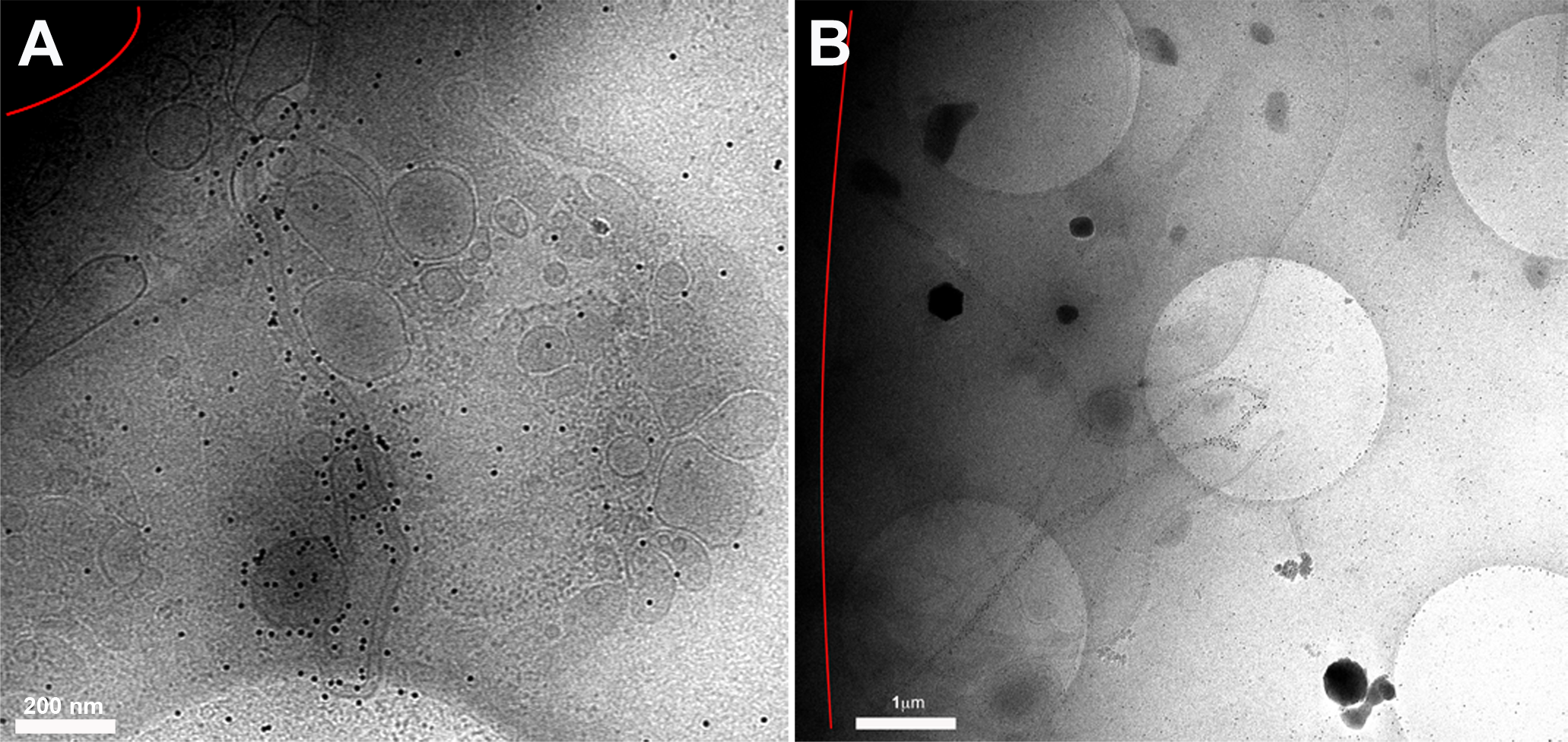

Supplement: Figure S5 — Cryo Electron Microscopy of budding filaments and Archetti bodies at the cell surface. Cell associated filaments and Archetti bodies close to the cell edge (red line) were seen to be surrounded by vesicles and cell processes (A, B). (TIF) [file ppat.1003413.s005.tif]

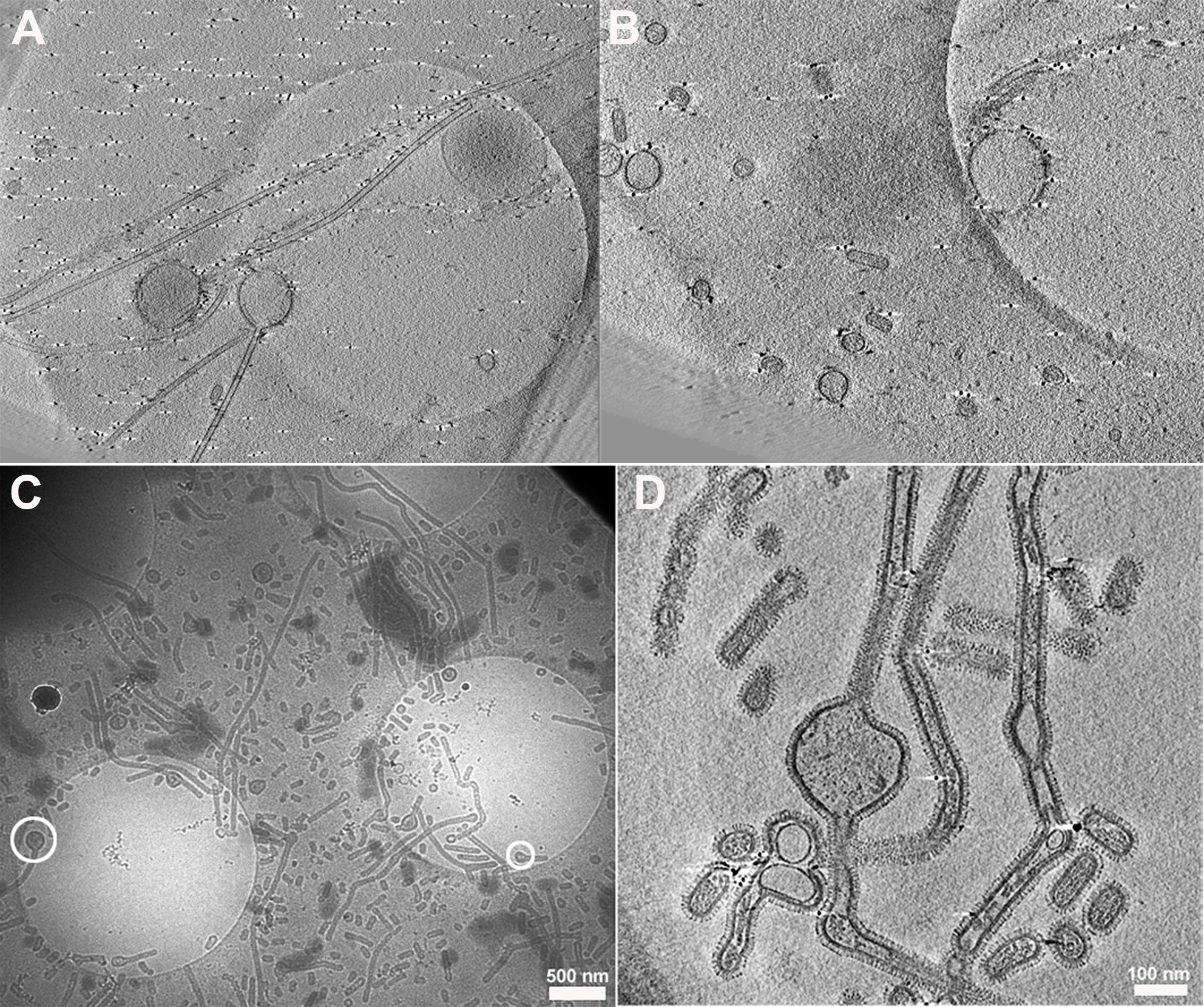

Supplement: Figure S6 — Visualising the pleomorphic structures of influenza A filaments and virions. In most tomograms of virus infected cells, small virions were not observed as they were most likely suspended in the culture media and did not adhere to the carbon support film. One tomogram was however recorded in which long filaments and Archetti bodies were seen (A) as well as some smaller virions (B). A low magnification (4000×) cryo image of purified virus reveals the extent of pleomorphism showing long filaments and a small number of Archetti bodies (white circles - C). Tomograms of purified virus however did not show Archetti bodies resembling those we saw in virus infected cells, rather filaments with varicosities along their lengths were seen that were less regular and frequently contained vacuolar structures (D). (TIF) [file ppat.1003413.s006.tif]

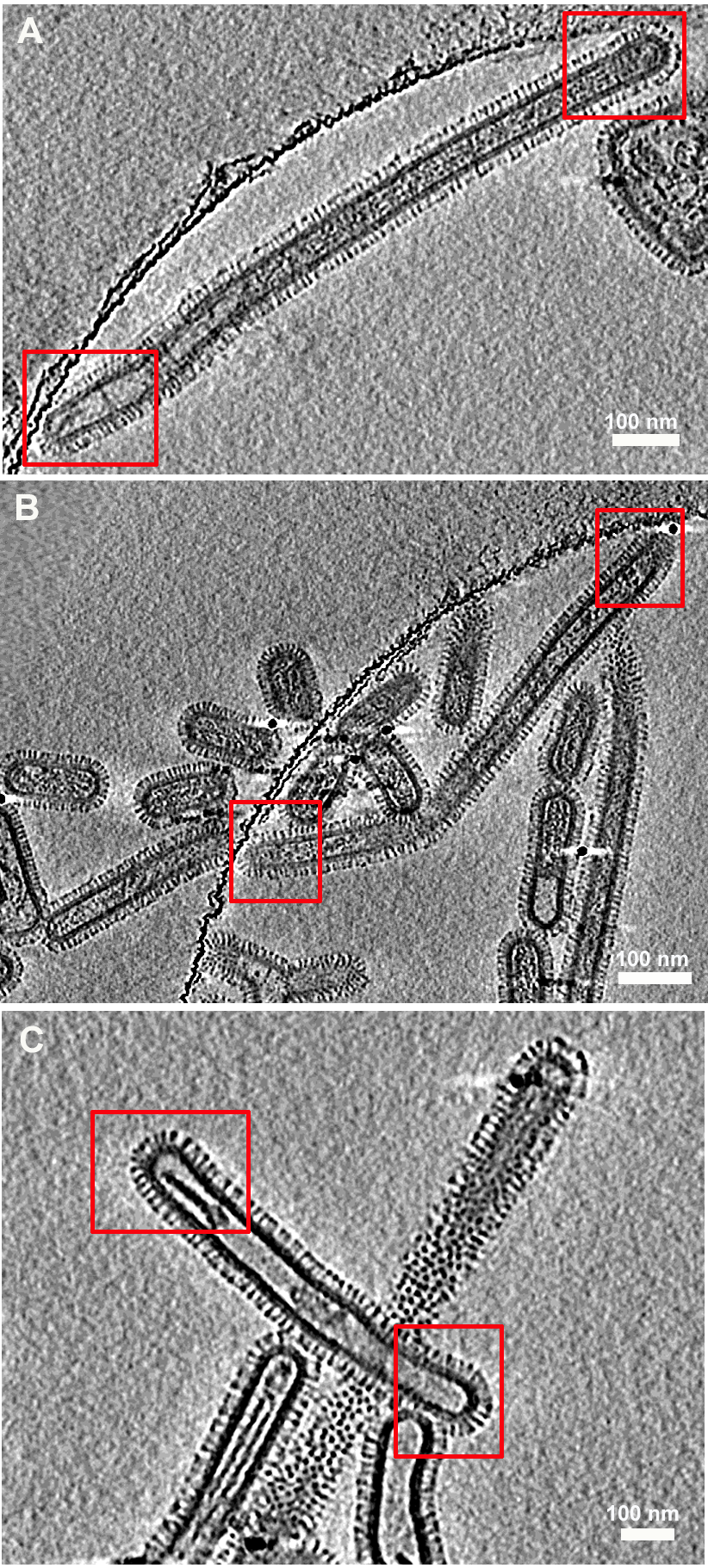

Supplement: Figure S7 — Cryotomography of purified influenza A filaments mostly did not have RNPs at their termini. Filaments in which both ends were visible were not commonly seen in tomograms owing to their great lengths however. Where such filaments were observed, the ends (red boxes) were not found to contain obvious RNP like density (A–C). (TIF) [file ppat.1003413.s007.tif]
